# Heat Shock Alters Mesenchymal Stem Cell Identity and Induces Premature Senescence

**DOI:** 10.3389/fcell.2020.565970

**Published:** 2020-09-22

**Authors:** Chen Shimoni, Myah Goldstein, Ivana Ribarski-Chorev, Iftach Schauten, Dana Nir, Carmit Strauss, Sharon Schlesinger

**Affiliations:** Department of Animal Sciences, The Robert H. Smith Faculty of Agriculture, Food, and Environment, The Hebrew University of Jerusalem, Rehovot, Israel

**Keywords:** bovine, mesenchymal stem cells, heat shock, oxidative stress, senescence, immunomodulation

## Abstract

Heat stress can have a serious impact on the health of both humans and animals. A major question is how heat stress affects normal development and differentiation at both the cellular and the organism levels. Here we use an *in vitro* experimental system to address how heat shock treatment influences the properties of bovine mesenchymal stem cells (MSCs)—multipotent progenitor cells—which are found in most tissues. Because cattle are sensitive to harsh external temperatures, studying the effects of heat shock on MSCs provides a unique platform to address cellular stress in a physiologically relevant model organism. Following isolation and characterization of MSCs from the cow’s umbilical cord, heat shock was induced either as a pulse (1 h) or continuously (3 days), and consequent effects on MSCs were characterized. Heat shock induced extensive phenotypic changes in MSCs and dramatically curtailed their capacity to proliferate and differentiate. These changes were associated with a partial arrest in the G1/S or G2/M checkpoints. Furthermore, MSCs lost their ability to resolve the inflammatory response of RAW macrophages in coculture. A possible explanation for this loss of function is the accumulation of reactive oxygen species and malfunction of the mitochondria in the treated cells. Heat shock treatments resulted in stress-induced premature senescence, affecting the MSCs’ ability to proliferate properly for many cell passages to follow. Exposure to elevated external temperatures leads to mitochondrial damage and oxidative stress, which in turn conveys critical changes in the proliferation, differentiation, and immunomodulatory phenotype of heat-stressed MSCs. A better understanding of the effect of heat shock on humans and animals may result in important health and economic benefits.

## Introduction

Elevated ambient temperatures are increasing in frequency and can severely affect human and farm animal well-being, especially during the summer period. Heat stress increases the concentration of intracellular reactive oxygen species (ROS) in cells (Slimen et al., 2014) and has detrimental effects on mammalian fertility and well-being (Kitagawa et al., 2004; Agarwal et al., 2014). ROS react with DNA, proteins, and other macromolecules, leading to an accumulation of mutations and misfolded proteins (Cooke et al., 2003). Moreover, increased ROS levels elicit stem cell depletion and functional defects in several tissues (Burgess et al., 2014). In bovine, the frequency of many chronic inflammation–related diseases is elevated during a hot period (Olde Riekerink et al., 2007; Chen S. et al., 2018), resulting in reduced animal welfare and significant economic losses to the dairy industry (Key et al., 2014). *In utero* heat stress was recently found to reduce the placental weight and blood flow and decrease birth weight of calves, and they impaired innate and cellular immunity (Dado-Senn et al., 2020). However, although heat is a common stressor, the functional connection between elevated temperatures and the higher rates of chronic inflammation is still obscure.

Adult stem cells are the longest living proliferative cells in multicellular organisms (Uccelli et al., 2008). They have an intrinsically increased risk of accumulating metabolic and genetic damage that will eventually lead to their destruction. The accumulation of such damage can be enhanced by extrinsic factors including environmental stress or exposure to toxins, together leading to the functional decline of the stem cells (Ermolaeva et al., 2018).

Mesenchymal stem/stromal cells (MSCs) are nonhematopoietic multipotent cells, most frequently derived from adult tissue sources such as bone marrow and adipose tissue or birth-associated tissue such as endometrial and placental tissues, amnion, and umbilical cord (Hass et al., 2011; Nowakowski et al., 2016). In the body, MSCs regulate bloodstream monocyte frequencies in reaction to inflammation (Mendez-Ferrer et al., 2010; Shi et al., 2011) and are capable of multilineage differentiation into cell types such as adipocytes, osteoblasts, chondrocytes, myocytes, β-pancreatic islets cells, and neuronal cells (Kuroda and Dezawa, 2014; Gao et al., 2016). *In vitro*, MSCs have been reported to exert anti-inflammatory, immunosuppressive, and trophic characteristics (Carrade et al., 2012; Kuroda and Dezawa, 2014; Li Y.W. et al., 2017; Shi et al., 2018). They exert these effects by influencing proliferation, recruitment, function, and fate of both the innate and adaptive immune cells, including T cells, B cells, dendritic cells, natural killer cells, and macrophages, which is likely mediated through both direct cell-to-cell contact and secretion of diverse immunoregulatory mediators (Aggarwal and Pittenger, 2005; Squillaro et al., 2015).

Several studies examining the cross-talk between MSCs and macrophages have shown MSCs to promote the anti-inflammatory M2 phenotype, which contributes to inflammation resolution and tissue repair in a variety of species (Braza et al., 2016; Chiossone et al., 2016; Yin et al., 2017). This results through the secretion of anti-inflammatory cytokines such as transforming growth factor β and TNF-stimulated gene 6 (Braza et al., 2016; Chiossone et al., 2016; Luz-Crawford et al., 2016; Howie et al., 2017; Li Y.W. et al., 2017). MSCs under intrinsic or extrinsic induced damage might become senescent (de Magalhaes and Passos, 2018). Senescent cells stop proliferating, become enlarged, and change their transcriptional pattern. Moreover, senescent cells secrete proinflammatory cytokines such as interleukin 1 (IL-1), IL-6, and IL-8 under control of the transcription factor nuclear factor κB [senescence-associated secretory phenotype (SAPS)] (Coppe et al., 2008). These stabilize senescence in an autocrine fashion and contribute to bystander effects, i.e., induction of DNA damage and senescence in normal surrounding cells (Gorgoulis et al., 2019). The entry into the senescent state is the result of either replicative senescence (cellular aging) or stress-induced premature senescence (SIPS) (Toussaint et al., 2002). SIPS is a state of irreversible division arrest of cells that retain their metabolic activity and is caused by persistent exposure to environmental stress, namely, heat stress, oxidative stress, or DNA-damaging mediators (Glotin et al., 2008; Alekseenko et al., 2012). Although much is known about aging-related stress, the causes and consequences of SIPS, as well as the degree and significance of SIPS in physiological conditions, are not well known and understood.

The objectives of this study are to characterize the immunomodulatory properties of bovine umbilical cord (bUC) MSCs and to examine the effect of high temperature [e.g., heat shock (HS)] on their function. MSCs of bovine origin have been previously isolated and characterized from different tissues (Bosnakovski et al., 2005; Cabezas et al., 2014; Mançanares et al., 2015; Xiong et al., 2016). *In utero* thermoregulation is dependent on the mother’s core temperature, and maternal heat stress can impact the fetal temperature through the fetal–placental circulation (Dado-Senn et al., 2020). Therefore, we investigated the properties of bUC-MSCs that survived physiological HS exposure for varying periods of time and under a range of temperatures. We show that while sublethal temperature shock induced SIPS and impaired bUC-MSC capacity for differentiation into multiple cell lineages, the effect on immunomodulatory functions is dependent on the duration of the HS.

## Materials and Methods

### Tissue Processing and Cell Culture

The UC tissue was processed following Toupadakis et al. (2010). Cells were plated in a low-glucose Dulbecco modified eagle medium (Gibco, Carlsbad, CA, United States) containing 10% fetal bovine serum (Gibco) and a penicillin–streptomycin mixture (3%), expanded, and cryopreserved at different passages. For more details, see Supplementary Material and Methods.

### Population Doubling Time Assessment

Following pulse and constant HS treatments, 100 K cells from each treatment were plated in 10-cm plates. This process was repeated every 4–6 days for 15 passages (over 100 days). Population doubling (PD) and PD time were calculated using the formulas *N* = N0 × 2 d (where N, N0, and d are the final cell numbers, the initial cell number, and the number of cell divisions, respectively) and *N* = N0 × 2t/τ (where *N*, N0, and τ are the final cell numbers, the initial cell number, and PD time, respectively).

### Evaluating Immunomodulation Function

Mesenchymal stem/stromal cells’ anti-inflammatory properties were examined through their ability to suppress the immune response, testing their effect on T-cell proliferation using a mixed lymphocyte reaction [adapted from Arzi et al. (2017)] and examining phenotypic shifts in macrophages toward their anti-inflammatory state (M2) [adapted from Chiossone et al. (2016), Luz-Crawford et al. (2016), Yin et al. (2017)]. For more details, see Supplementary Material and Methods.

### Oxidative Stress Levels Detection

We used a CellROX Green Reagent (Invitrogen, Carlsbad, CA, United States) following the manufacturer’s instructions, with or without 500 μM H_2_O_2_ (Sigma, St. Louis, MO, United States) for 30 min at 37°C. Three biological repeats were used for each treatment. For more details, see Supplementary Material and Methods.

### Mitochondrial Membrane Potential Measurement

We used a JC-1 assay (ENZO Life Sciences International, Farmingdale, NY, United States) as described by Smiley et al. (1991). Mitochondrial membrane potential (MMP) was qualified based on the fluorescence emitted and classified into two main colors: red—high potential, and green—low potential. Three biological repeats were prepared for each treatment, and a 1-h prestaining incubation with 500 μM H_2_O_2_ was prepared as a positive control. For more details, see Supplementary Material and Methods.

### Cell Proliferation Assay

We used a CellTrace carboxyfluorescein succinimidyl ester (CFSE) Cell Proliferation Kit (Invitrogen). Two to three biological repeats were used for each treatment. For more details, see Supplementary Material and Methods.

### Cell Cycle Analysis

Following pulse and constant HS treatments, full cell cycle analysis and S phase quantification were carried out using PI/BrdU stainings adapted from Lehner et al. (2011). Immunohistochemistry was performed as described previously (Lichter et al., 1990; Selig et al., 1992). More than 100 cells were counted in each slide. For more details, see Supplementary Material and Methods. For detection of S-positive cells during the 43 h prior to the end of HS treatments, cells from 42°C pulse HS and 72-h constant HS were incubated with BrdU for a range of time periods (1–43 h) and taken for BrdU detection protocol on day 3. Three biological repeats were taken for each treatment, and a negative control was taken without BrdU, but with first and secondary antibodies.

### Senescence-Associated β-Galactosidase Marker Assay

This was carried out using a Senescence Assay Kit (Abcam, Cambridge, United Kingdom) and measured by flow cytometry. Three biological repeats were used for each treatment.

### Statistical Analysis

GraphPad Prism (La Jolla, CA, United States) was used for statistical analysis and visualization. *P* < 0.05 was considered statistically significant.

## Results

### MSC Culture From Cow Umbilical Cord

**FIGURE 1:**
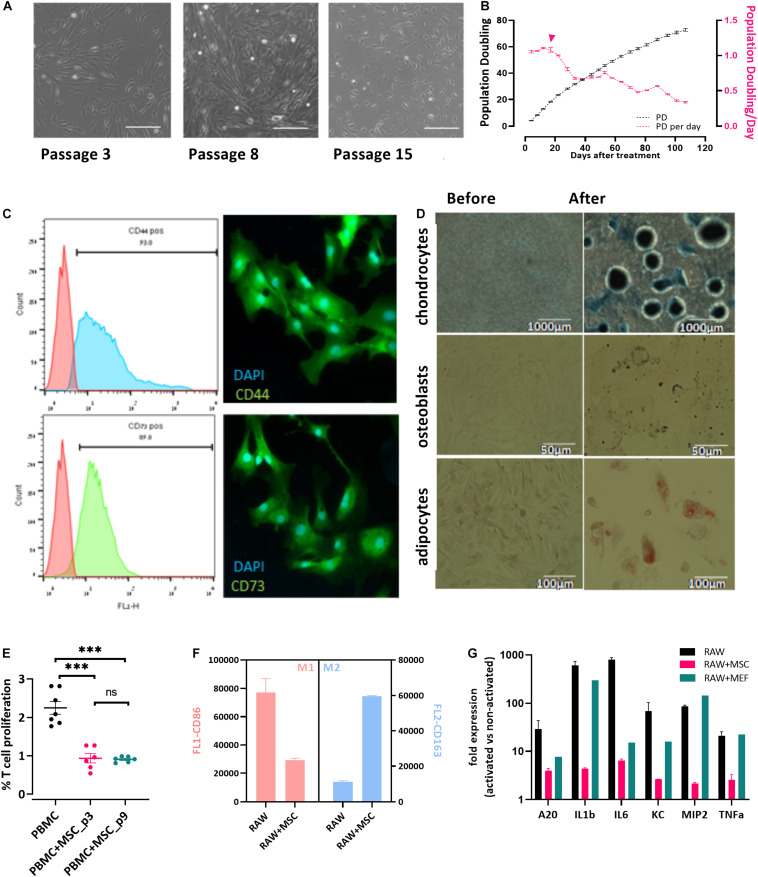
Cells isolated from cow’s umbilical cord display known MSC characteristics. **(A)** bUC-MSC displays a spindle-shaped morphology, proliferates, and adheres to plastic surfaces. No changes in cell morphology were observed for up to eight passages, but at passage 15, proliferation slowed. Scale bar = 500 μm. **(B)** bUC-MSC total population doubling (PD, black line) and population doubling time (PDt, pink line) for 25 passages. Population doubling was calculated as described in the “Materials and Methods” section. **(C)** Immunophenotyping (by flow analysis, left panel) and fluorescent microscopy imaging (right panel, with DAPI staining for nucleic acid, scale bars = 50 μm) of CD44 and CD73 MSC markers. **(D)** bUC-MSCs can differentiate into chondrocytes, osteoblasts, and adipocytes (stained by Alcian blue, Alizarin red, and oil red O, respectively). **(E)** T-cell proliferation assays were performed using bovine peripheral blood mononuclear cell (PBMC) activated by Con-A and cocultured with bUC-MSCs for 4 days at a MSC: PBMC ratio of 1:5. Cell proliferation was determined by MTT assay. The results represent the fold change between the Con-A–activated and nonactivated PBMCs, measured by the absorbance at 540 nm, and the activation of PBMC alone was set to 1. Data are mean ± SEM for *n* = 6, and Student *t*-test was used for comparison of means. ***p < 0.001. **(F)** Macrophages cell line (RAW) was cocultured with bUC-MSCs for 21 h and then activated by LPS for 3 h. RAW cells without bUC-MSCs were examined as reference cultures. RAW cells were then stained and flow-analyzed with proinflammatory M1 (pink bars, CD86) or anti-inflammatory M2 (blue bars, CD163) distinctive antibody. Mean fluorescence intensity ± SEM is indicated for *n* = 3. All bars are significantly different (*p* < 0.01) by Student *t*-test. **(G)** The transcriptional profile of five mouse genes was investigated by RT-qPCR on RNA extracted from the activated macrophages with or without cocultured bUC-MSC or MEF negative control. The expression levels of typical proinflammatory genes were reduced in RAW cells cocultured with bUC-MSCs but not with MEFs. Three housekeeping genes were used as reference genes—UBC, EIF5a, and EEF1A1. Data are mean ± SEM for *n* = 3 of RAW, RAW + MSC, and *n* = 2 for RAW + MEFs.

Bovine umbilical cord-Mesenchymal stem cells were isolated from bUCs as described in “Materials and Methods” and cultivated for 15 cell passages. The cells exhibited typical fibroblastic morphology (Figure 1A) and maintained proliferation potential for at least 15 passages (over 100 days, Figure 1B). It should be noted, however, that after the fifth passage the number of populations doubling per day was reduced significantly (Figure 1B, pink line and error head). Following isolation, the cells were characterized as bUC-MSCs by marker expression (Figure 1C), and chondrogenic, osteogenic, and adipogenic differentiation potential was assessed (Figure 1D).

### Bovine Umbilical Cord-Mesenchymal Stem Cells Have Immunomodulatory Potential

Following the initial characterization of the bUC cells as MSCs, we examined their immunomodulatory potential as suggested by others (Menard et al., 2013). First, inhibition of T-cell proliferation was examined. PBMCs were isolated from whole blood and cocultured with bUC-MSCs for 4 days at a ratio of 1:5, respectively, with or without activation with concanavalin A (Con-A). T-cell proliferation was assessed by MTT assay, and a fold change between activated and nonactivated cells for T cells alone or cocultured with bUC-MSCs is presented in Figure 1E. While activated T cells alone exhibit proliferation, coculturing with bUC-MSCs reduced T-cell proliferation significantly. The effect remained strong even when cells were at passage nine.

Next, because the influence of MSCs on T cells is thought to be modulated at least in part by macrophages, the effect of bUC-MSCs on the balance between proinflammatory M1 macrophages and anti-inflammatory M2 macrophages was assessed (Francois et al., 2012). RAW (mouse macrophages cell line) cells were cocultured with bUC-MSCs for 21 h in normal RPMI media, which was then changed to conditioned RPMI media with LPS for 3 h for macrophage activation. Following the 24-h coculture, the identity of the cells was examined by flow cytometry using CD86 and CD163 antibodies (Figure 1F) and by reverse transcriptase–quantitative polymerase chain reaction (RT-qPCR) for cytokine expression levels (Figure 1G). As expected, activated RAW cells present a high degree of CD86+ M1 type, but once cocultured with bUC-MSCs, the degree of CD86 expression was significantly reduced, whereas CD163 expression inclined. The coculture with MSCs reduced the expression of proinflammatory cytokines such as IL-1β and tumor necrosis factor α (TNF-α), an effect that the control cells (mouse embryonic fibroblasts-MEFs) did not demonstrate. These results establish the immunomodulatory capacity that the bUC-MSCs hold in suppressing inflammation.

### Thermal Preconditioning Affects Cell Growth and Morphology

**FIGURE 2:**
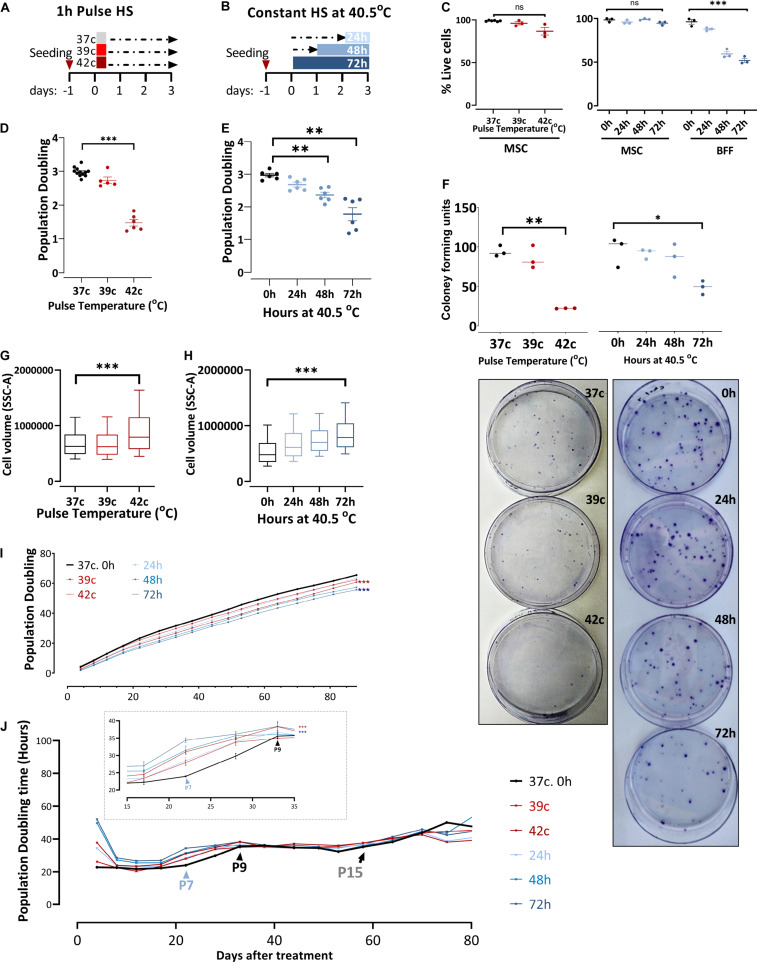
In response to heat shock, bUC-MSCs change their morphology and proliferation rate. **(A)** Pulse-HS: bUC-MSCs are plated and 24 h later are placed at 39 and 42°C for 1 h and then allowed to recover at 37°C for 3 days. **(B)** Constant HS: bUC-MSCs are plated and 24 to 72 h later are moved to grow at 40.5°C for 1–3 days. **(C)** Dead: live MSC ratio as measured using trypan blue does not change dramatically following both HS treatments. In contrast, bovine fetal fibroblast (BFF) shows massive death rates following constant HS treatment. **(D)** bUC-MSC Population doubling declines following pulse and **(E)** constant HS. **(F)** Drop in clonogenicity of cells after pulse or constant HS. Data from C to F are mean ± SEM for *n* ≥ 3. Unpaired, two-tailed Student *t*-test was used for comparison of means. ***p < 0.001, **p < 0.01, *p < 0.05, ns = nonsignificant. **(G)** Side-scatter geo-mean data of cells after pulse and **(H)** constant HS are shown. One flow analysis of 10,000 cells is shown (representative result, *n* = 3). **(I)** Growth curve of untreated cells (black line) and cells after HS. Each line represents a sample, and each time point represents a passage. The mixed-effect model was used for comparison, ***p < 0.001. **(J)** Population doubling time measured by hours shows the kinetics of proliferation/passage. Immediately after the HS treatment, the cells have nearly ceased to proliferate, but after 1 passage, they narrow the gap with the control cells. Inset shows a narrower window of 20 days between passages 6 and 9. However, the HS cells never completely closed the gap, and even after about 15 passages, all cells are declining toward replicative senescence and cease proliferating. Mixed-effect model was used for comparison, ***p < 0.001.

To examine the effect of changing external conditions on the potency of the cells, we evaluated the HS response of bovine MSCs. HS treatment in tissue culture was performed by applying two protocols meant to examine the short- or long-term consequence of exposure to physiological sublethal HS conditions. For pulse HS, MSCs at early passages (P1–P4) were moved from 37°C to either 39°C or 42°C for 1-h HS followed by a 3-day recovery back to 37°C (Figure 2A). For the constant HS protocol, cells were moved from 37 to 40.5°C for 24, 48, or 72 h (Figure 2B). Constant and pulse HS protocols were performed, and the effect on the cells’ survival, growth, clonogenicity, and morphology were examined. No significant death was observed in the MSC culture using trypan blue staining (Figure 2C), yet the number of cells was lower following the HS treatment. This behavior, also noted and studied previously in the context of MSCs (Antebi et al., 2018), is not representative of all bovine fibroblasts. When bovine fetal fibroblast cultures were treated with similar HS protocols, the majority of the cells died (Figure 2C).

Enhanced ability of proliferation is a well-known trait of multipotent stem cells, and bUC-MSCs have shown the ability to proliferate for multiple passages (Figure 1B). However, following severe pulse or constant HS, PD declined from ∼3 PD in the 4 days since plating to 1–2 PD in the cells that suffered the HS (Figures 2D,E). Additionally, the reduced clonogenic capacity of the HS-treated cells was evident by significantly lower colony-forming unit fibroblasts (CFU-f) numbers as compared to MSCs grown in normothermia (Figure 2F, a graph summarizing triplicate results and representative pictures of the plates). This is in agreement with studies showing that different stresses might cause a decrease in the number of CFU-f of MSC culture (Boyette et al., 2014; Kheirandish et al., 2017). These results indicate that induced HS reduces colony-forming capability and cell division in culture without increased cell death. Furthermore, the treated cells became flattened and larger (morphology typical of senescent cells) as evident under the microscope (Supplementary Figures S1A,B) and measured by flow analysis side scatter (Figures 2G,H).

To assess whether the change in the cells proliferative capacity is temporal or inherited to the daughter cells, we continued to grow the HS-treated cultures for 80 more days following the treatment (which took place on days 0–4, P2) and measured the cumulative PDs (Figure 2I). Although at each passage live cells were seeded at a fixed confluence (1 × 10^5^ cells/9.6 cm^2^), the gap between the control and HS-treated cells remained stable for more than 13 passages. By following the attenuation of the number of doublings per day (PDt), we noted that the cells’ proliferation capacity almost fully recovered after their immediate reduction following the HS treatments [as in Wiese et al. (2019)]. Approximately three passages later (P7, blue arrowhead in Figure 2J, inset), the treated cells started slowing their PDt, whereas the control culture PDt started to decrease only two passages afterward (P9, black arrowhead in Figure 2J, inset). This finding suggests that the treated cells initiated cell cycle slow-down and replicative senescence earlier than the untreated MSCs. However, all cells continued proliferation at a slow but stable rate for more than 15 passages (black arrow in Figure 2J) before the culture became inhomogeneous and eventually ceased proliferation completely.

### Functional Consequences of HS

**FIGURE 3:**
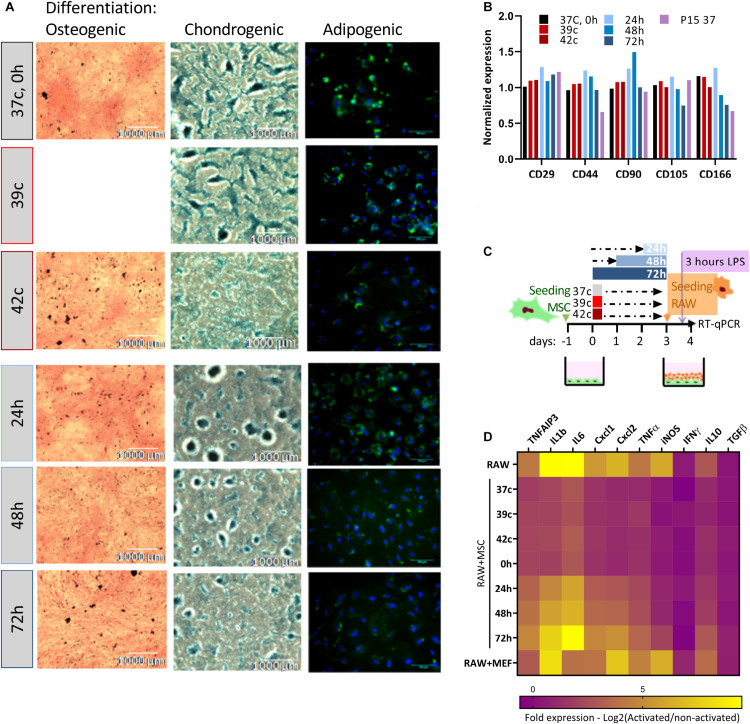
Loss of differentiation and immunomodulation abilities following heat shock. **(A)** bUC-MSCs gradually lose the ability to differentiate into osteocyetes (left, stained by Alizarin red), chondrocytes (middle, stained by Alcian blue), and adipocytes (right, stained by Bodipy and DAPI) following pulse and constant HS. Representative pictures from three independent experiments are shown. **(B)** No change in expression levels of typical MSC markers following HS, with all samples normalized to control untreated MSCs. **(C)** Macrophages cell line (RAW, illustrated orange) was cocultured with bUC-MSCs (in green) for 21 h following HS treatment and then activated by LPS (purple) for 3 h. RAW cells without bUC-MSCs were examined as a reference, and coculture with neutral cells (MEFs) was carried out to control for coculture effect. **(D)** The transcriptional profile of 10 mouse genes was examined by RT-qPCR. The expression levels of typical proinflammatory genes were high (yellow) in RAW cells alone and reduced (violet) in RAW cells cocultured with bUC-MSCs but not with MEFs. Long HS treatment at 40.5°C eliminated the reduction, indicating that those cells were malfunctioning. Three housekeeping genes were used as reference genes—UBC, EIF5a, and EEF1A1. Data are the mean for *n* = 3.

Together with proliferation capacity, immunophenotypic analysis and three-lineages differentiation are the parameters for the characterization of MSCs (Dominici et al., 2006). We analyzed how the *in vitro* differentiation potential is affected by the HS treatment (Figure 3A). Osteogenic, chondrogenic, and adipogenic differentiation potential declined following the severe pulse and constant HS. Osteogenic and chondrogenic differentiation of HS-treated cells were impaired mainly for 42^*o*^C, 48- and 72-h treatments. As fewer differentiation centers were visible, the intensity of staining was weaker, and their size was smaller. HS-treated cells exhibited similar results following adipogenic differentiation. Delayed fat accumulation was visible, the morphologic change took longer to initiate, and fewer cells accumulated lipid droplets that were also smaller as compared to control cells. These results suggest that even short thermal stress has an incremental impact on the overall differentiation potential of MSCs *in vitro*, which might explain some of the impaired physiological functions of animals exposed to heat stress. The immunophenotypic analysis showed that although the composition and expression level of surface markers change during long-term cultivation (Wagner et al., 2008), no effect of the HS treatments on the expression levels of the distinctive surface markers was observed (Figure 3B).

### The Functional Consequence of HS on MSC Immunomodulatory Capability

The effect of MSCs on the immune system is a major reason for the interest in these cells and an important characteristic for both therapeutic use and physiological homeostasis. We analyzed the capability of MSC coculture to induce the switch of activated macrophages from proinflammatory M1 to anti-inflammatory M2 by comparing the cytokine expression pattern of RAW macrophages cocultured with MSCs before and after HS treatments (as illustrated in Figure 3C). While the coculture of RAW with normal MSCs or MSCs after the pulse HS treatment reduces the expression of mouse proinflammatory cytokines such as KClike Cxcl1, IL-1b, and TNF-α, the constant HS treatment eliminates this immunomodulatory capacity of the cells (Figure 3D, see also Supplementary Figure S2).

Together, these data indicate a dissimilar effect of the two HS protocols, at least in some aspects. While short pulse HS impairs proliferation and differentiation, but not immunomodulation, constant thermal stress of 24 h or longer can reduce the immunomodulation capability of the surviving MSCs. In a physiological context, this may suggest that the lack of regulation on the immune system is the basis for the higher probability of chronic inflammations. Accordingly, we looked to uncover the mechanism for this loss-of-stemness and immunomodulatory functions in the stressed cells.

### HS Increases Intracellular ROS Production in Cell Culture

**FIGURE 4:**
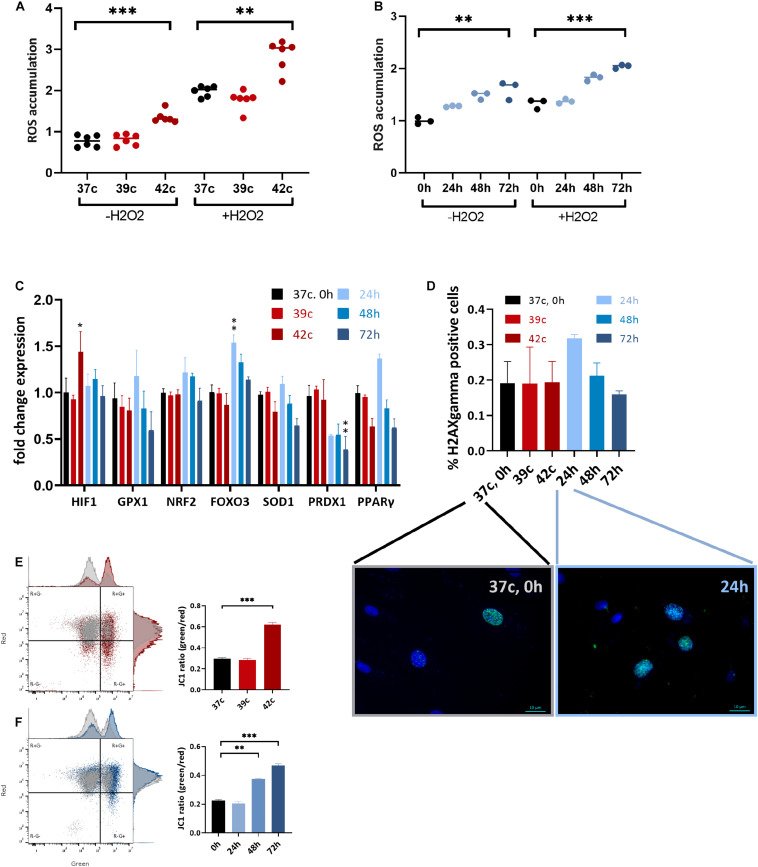
Change in metabolic function following HS in bUC-MSCs. Flow cytometry results of CellROX staining for **(A)** cellular ROS levels after pulse HS without and with 500 μM H_2_O_2_ for 30 min. **(B)** cellular ROS levels after constant HS without and with 500 μM H_2_O_2_ for 30 min. Data presented are a mean of green fluorescence, ±SD *n* = 6. **(C)** Change in expression levels of oxidative stress response pathway genes. All genes were normalized to control bUC-MSCs. Statistical significance was determined using the Bonferroni–Sidak method, and results represent the mean ± SEM, *n* = 4. **(D)** Histone H2AX phosphorylation was measured by fluorescent microscopy imaging with anti-γH2AX (green) and DAPI staining for nucleic acid (blue), scale bars = 10 μm. Negative and positive nuclei were counted (∼200 from each slide) from two unrelated experiments, mean ± SEM are shown. **(E)** Membrane potential was measured using JC-1 assay and flow analysis (see example in the left panels). Percent of red cells (upper left, R+G–) declined after pulse HS (red) and **(F)** constant HS (Blue) as compared to the untreated cells (gray), *n* = 3. Statistics: two-way ANOVA with Benjamini, Krieger, and Yekutieli *post hoc* test as compared to 37°C, 0 h, **p < 0. 01, ***p < 0.001.

HS has been implicated in promoting oxidative stress either through excessive ROS production or decreased antioxidant defense, as was previously shown in dairy cows (Bernabucci et al., 2002; Abdelnour et al., 2019). To examine whether HS affects the levels of oxidative stress in MSC cell culture, CellROX dye levels were examined at day 3 after HS, with or without the addition of H_2_O_2_ for the last 30 min. Interestingly, 1 h at up to 39°C did not affect the ROS levels, whereas 42°C for 1 h induced high oxidative stress even after a 3-day recovery (Figure 4A). Similarly, a direct positive correlation was found between the number of days at 40.5°C and ROS levels (Figure 4B). The addition of H_2_O_2_ in the last 30 min was made to examine the resistance of the cells to secondary oxidative stress. However, although the net ROS levels were elevated, the positive correlation between ROS levels and HS remained, indicating that acclimation to HS did not involve resistance to secondary oxidative stress. This behavior is specific to MSCs after HS because the cells did not change their morphology (Supplementary Figure S3A) or ROS levels (Supplementary Figure S3B) when grown in 1% oxygen—hypoxia—for 3 days.

Expression of some stress response–related genes (FOXO3, GPX1, and PPARγ) was elevated following 24-h HS, but not in other HS-treated cells despite the elevated ROS levels these cells contained (Figure 4C). Hypoxia-inducible factor α (HIF1α) was slightly up-regulated 3 days after the 1-h pulse HS but not after the constant HS. Additionally, no significant histone H2AX phosphorylation—a measure of DNA double-strand breaks—could be identified in the cells after the HS treatment by immunofluorescence detection of γH2AX (Figure 4D). We hypothesize that the increase in DNA damage loci after 24 h in HS (Figure 4D, graph and representative pictures) is resolved in the later constant HS time points—48 and 72 h—by the action of the antioxidant gene product transcribed at 24 h (Figure 4C). Thus, although the HS is followed by higher oxidative stress in the cells, no major DNA damage was caused or accumulated. This is in line with the minor proportion of cell death and suggests that the notable drop in the number of cells following treatment is not due to severe damage.

Functioning mitochondria is required for the immunomodulation activity of MSCs (Islam et al., 2012). To examine if the HS treatment impaired the mitochondria, the JC-1 assay for the detection of MMP was used (Smiley et al., 1991). JC-1 indicates high MMP by the formation of red fluorescent aggregates, while at the same time displays MMP depolarization by forming a green fluorescent monomer. Normal untreated cells show a low green/red ratio (25% of the cells contain damaged mitochondria), whereas an average of 62% of the pulse (Figure 4E) and 46% of the constant (Figure 4F) HS cells present green, depolarized membrane potential. This decline in mitochondrial activity may be the cause of the drop in PD and reduced immunomodulation in the treated cells.

To examine if elevated ROS levels and mitochondrial damage are inherited through passaging, we stained the same stressed cells with CellROX dye after long passaging at P15 (Supplementary Figure S3C). At this stage, cells show slower growth (Figures 2J,I) and 10 times higher ROS levels than at P3. However, a small difference remains between the previously HS-treated cells and the control cells. In contrast, mitochondrial damage is elevated in all aged cells, but no difference could be observed between the previously HS-treated cells and the control cells (Supplementary Figure S3D). Overall, these results show that cells after either long-term culturing or HS show parallel metabolic and functional attenuations.

### HS Leads to Decreased Proliferation, Cell Cycle Arrest, and Premature Senescence

**FIGURE 5:**
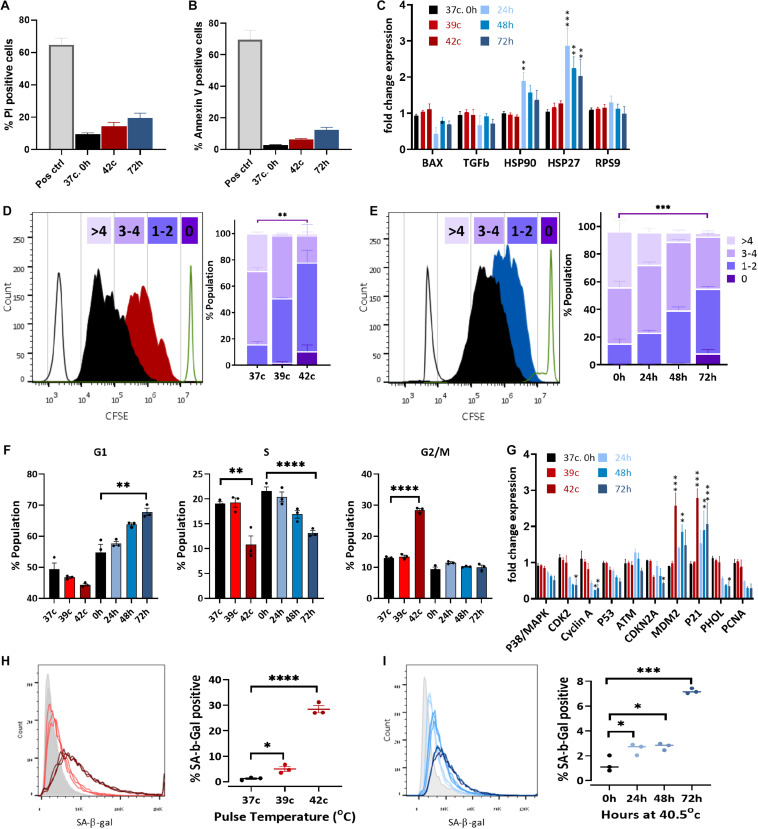
Heat shock induces cell cycle arrest and premature stress-induced senescence (SIPS). Flow cytometry assay of cell death using PI staining **(A)** and apoptosis with annexin V staining **(B)** of pulse HS at 42°C and constant HS for 72 h. **(C)** Change in expression levels of heat shock proteins and apoptotic-related genes. RPS9 was used as a control gene, and data were normalized to WT bUC-MSC expression levels. Statistical significance was determined using the Holm–Sidak method; results represent the mean ± SEM, *n* = 4. **(D,E)** Cell proliferation was assessed by the CFSE dilution method following pulse HS **(D)** or constant HS **(E)**. Each experiment was performed in triplicate; results represent the mean ± SD. Statistics: two-way ANOVA with Benjamini, Krieger, and Yekutieli *post hoc* test as compared to 37°C, 0 h, **p < 0.01, ***p < 0.001. **(F)** PI/BrdU staining flow cytometry analysis of cell cycle phases distribution following constant or pulse HS. Data are presented as mean ± SEM. Statistics: *t*-test with the Holm–Sidak method, *n* = 3–9. **(G)** Detection of BrdU incorporated for 1–43 h into the DNA of pulse HS at 42°C and constant HS for 72 h treated cells by flow cytometry. Data are the mean ± SEM for n = 3–6. **(H)** Change in expression levels of cell cycle–related genes. Data were normalized to WT bUC-MSC expression levels. Statistical significance was determined using the Holm–Sidak method; results represent the mean ± SEM, *n* = 4. **(H,I)** % cells positive for SA-β-gal staining during cell recovery from pulse **(H)** and constant HS **(I)**. Data are mean ± SEM for *n* = 6 and a Student *t*-test was used for comparison of means, ***p < 0.001, ****p < 0.0001.

A significant decrease in cell growth following elevated ROS levels and mitochondrial dysfunction could indicate either elevated apoptosis levels or changes in the proliferation capacity of the cells due to cell cycle arrest or slow-down. To examine the first option, we measured the percentage of dead and apoptotic cells in the population, staining for propidium iodide (PI) or annexin V, respectively. The two most extreme HS conditions used—42°C for 1 h and the 72-h HS—had a twofold increase in dead cells (from 12 to 21, and 27%, respectively, Figure 5A and Supplementary Figure S4A). A minor increase in apoptosis from about 5 to 6, or 12% of the cells being positive to annexin V was also detected (Figure 5B). This increase is very small and could not explain the reduction in cell numbers after HS. Furthermore, no change in the apoptosis-related gene BAX was observed after any of the HS treatments (Figure 5C). Hence, we examined a second option to assess changes in cell proliferation rate.

The expression of HS proteins HSP27 and HSP90 increased significantly following constant HS (Figure 5C), which indicates acclimation to heat in these cells (Matozaki et al., 2019).

In order to examine the proliferation rate of bUC-MSCs following HS treatments, a CFSE fluorescent tracing, diluted at each cell division, allowed us to visualize and distinguish between cell generations. CFSE staining was performed on bUC-MSCs on day 0, followed by pulse or constant HS experiments, and the fluorescent signal was measured by flow cytometry on day 3. With each cell doubling, the cell staining became weaker. The control cells, grown at 37°C, doubled three to five times during these 4 days (Figures 5D,E—black histogram). After pulse HS at 42°C, a significant decrease in cell doubling was observed, and about 10% of the population did not complete more than one round of doubling (Figure 5D). Following constant HS, a significant decrease in cell doubling was observed again, with a high variation in the number of doubling times in the population (Figure 5E). These results signify a decrease in proliferation rate after constant HS as well as following a 1-h pulse of high temperature, which affects the cell cycle even after a 3-days recovery in normothermia.

Next, PI-BrdU flow cytometry analysis revealed that following pulse HS, cell cycle was arrested at G2/M (Figure 5F and Supplementary Figure S4B top panels), whereas constant HS led to G1/S arrest (Figure 5F and Supplementary Figure S4B bottom panels). To verify the reduction in % S phase, cells were similarly stained with anti-BrdU and S phase BrdU-positive cells were counted under the microscope (Supplementary Figure S4C) and by flow cytometer (Supplementary Figure S4D). The results show that, indeed, both pulse and constant HS cell populations are less replicating than the control untreated cells (Figure 5F and Supplementary Figures S4C,D). These results can point to two different situations: either homogenous populations with a longer cell cycle or two subpopulations of cells emerging from the HS treatment, of which is arrested in senescence or quiescence and the other showing normal proliferation capacity. To distinguish between these two options, cells were incubated with BrdU for a range of times before the HS protocol ended (Supplementary Figure S4E). Interestingly, the pulse HS treatment displayed a Gompertzian-like BrdU incorporation kinetics, with 5 to 15% fewer cells incorporating BrdU than the untreated population. The constant 72-h HS, however, presented a unique curve shape of BrdU incorporation, which may suggest a population-wide change in the kinetics of entering S phase. Transcriptional analysis of cell cycle–related genes revealed a significant increase in the expression of *CDKN1a* (*p21*), a cell cycle inhibitor, and major regulator of the senescence program (Alekseenko et al., 2014) (Figure 5G). In agreement with the absence of detectable DNA damage in the senescent population, *p53* and *ATM* are not up-regulated. The elevated expression of MDM2, which promotes *p53* degradation, might explain the low levels of apoptosis in the stressed populations. No *p16* (*CDKN2A*) upregulation is seen, in agreement with the previous findings (Dulić et al., 2000; Alekseenko et al., 2012; Muñoz-Espín et al., 2013; Zhai et al., 2019), suggesting this is not a requisite marker for senescence. Interestingly, S phase genes such as *PCNA* and *POLH* are down-regulated in the constant HS group, arrested at G1. Correspondingly, these genes are not down-regulated in the pulse HS cells that are arrested in G2/M.

This is the only gene that showed an increase following all the constant HS treatments, as well as in the pulse HS of 42°C, regardless of the recovery period at 37°C.

To dissect the long-term changes in the cells following HS, we compared the stressed cells (at P3) before and after culturing them for >10 additional passages (at P15) (Supplementary Figure S5A). JC-1 assay shows reduced mitochondrial potential in all aged cells, with no significant long term effect on the HS-treated cells (Supplementary Figure S5B). To examine if elevated ROS levels and mitochondrial damage are inherited through passaging, we stained the same stressed cells with CellROX dye after long passaging—at P15 (Supplementary Figure S5C). At this stage, cells show slowed growth (Figures 2J,I) and 10 times higher ROS levels than at P3. However, a small difference remains between the previously HS-treated cells and the control cells. In contrast, mitochondrial damage is elevated in all aged cells, but no difference could be observed between the previously HS-treated cells and the control cells (Supplementary Figure S5D). Overall, these results show that cells after either long-term culturing or HS show parallel metabolic and functional attenuations. We next compared the transcriptional program to examine the change in genes that are either typical MSC marker-, cell cycle-, or apoptosis-related genes and genes related to stress and inflammation (Supplementary Figure S5E). As expected, most genes had major changes in gene expression following long-term passages. *HSP90* presents down-regulation, similar to PPARγ, which was shown to decrease during cellular aging (Park et al., 2005; Ye et al., 2006; Li Y. et al., 2017). On the other hand, some genes presented upregulation in the P15 bUC-MSCs (*HIF1*, *GPX1*, *BAX*). However, no effect of past HS treatment was observed in the P15 cells, and all showed uniform transcription patterns. Interestingly, a cluster of cell cycle arrest–related genes [namely, *p38/MAPK*, *p53*, *CDKN2A* (*p16*) and *PRDX1*] with low expression levels in the aged P15 MSCs was similarly down-regulated following 72-h HS treatment and to a lesser extent also in the other constant and pulse HS-treated cells. However, some genes changed after HS but not at P15 (*p21, HSP27*) whereas others were up-regulated at P15 but not following HS (*BAX*, *GPX1*, and *NRF2*). This revealed that despite some similarities, the molecular mechanisms used following HS are not those turned on following replicative senescence.

Last, to examine whether cell cycle arrest following HS is a result of SIPS (Alekseenko et al., 2014), staining was performed with SA-β-Gal, which is a marker for detecting cellular senescence (Dimri et al., 1995). SA-β-Gal staining increased in the HS-treated cells both for the pulse (Figure 5H) and the constant (Figure 5I) HS protocols. When compared to P15 cells (Supplementary Figure S5B), SA-β-Gal staining for P4 cells after the pulse and constant HS showed much higher levels of senescence, confirming that mild HS indeed promotes senescence in bUC-MSCs.

## Discussion

We presented the effect of mild heat stress on the potency and function of bovine MSCs from the umbilical cord. Previous studies have shown that exposure to extremely high temperatures (45°C) for a very short time can lead to senescence of MSCs (Alekseenko et al., 2012, 2014) while inside the body the cells can hardly be exposed to this temperature. Other studies have shown an improvement in MSC survival and function following short and mild HS (Chen X. et al., 2018; McClain-Caldwell et al., 2018; Wang et al., 2019). Therefore, we examined the effect of slightly elevated temperatures (constant HS) on the bUC-MSCs to determine if this HS would affect their proliferation, differentiation, and immunomodulation ability. The different time points (24, 48, and 72 h) were meant to follow the development of cellular acclimation following a sublethal exposure to heat (Matozaki et al., 2019). We also examined whether a short elevation of temperature—still within the physiological range—will affect cells 3 days afterward (pulse HS). Although we tried to stay within the physiological range, it is not clear how many cells are exposed to such temperatures in the body and for how long.

We have shown that in many ways the 1-h pulse HS has a similar long-term effect to that of the 72-h constant HS. Although initially there is no substantial death, the cells express *p21* and slow down their cell cycle, and the differentiation efficiency is reduced. This might indicate that the detrimental effects of HS on the cells (elevated ROS, mitochondrial damage) initiate a chain of events that lead to the onset of SIPS, no matter the length of the HS. However, unlike the constant HS, the pulse HS protocol did not impair the immunomodulation ability of the cells. The different stages of cell cycle arrest and gene expression patterns may explain this discrepancy. In both HS treatments, the percentage of senescent cells was never greater than 30%, and the remaining cells displayed almost normal features after passaging. However, even a low percentage of senescent cells in the population was shown to cause adverse consequences, supporting the need to study the effect of small environmental changes on the cellular population.

It could be hypothesized that in a subpopulation of the cells, the HS caused a shift to a state of reversible quiescent and not to irreversible senescence. Supporting this hypothesis, the BrdU long incubation displays the classical Gompertzian-like kinetics for the WT and the pulse HS cells, as expected with equal cell cycle durations, whereas the constant HS curve varies greatly. Hence, it seems that a subpopulation of the 72-h HS–treated cells present low self-renewal rates, e.g., quiescence. This state might present an advantage to the organism because the cells will be more protected and less metabolically active in times of thermal stress, but retain the opportunity to be activated and function as normal stem cells in times of need. Indeed, to allow time for DNA repair following oxidative damage, the cells activate their cell cycle checkpoints, leading to cell cycle arrest and preventing the replication of damaged and defective DNA (Dizdaroglu, 1992). On the other hand, oxidative stress, as expressed by elevated levels of cellular ROS and decreased MMP, is a well-known implication of heat stress (Bernabucci et al., 2002) and inducer of SIPS in many cells (Dasari et al., 2006; Glotin et al., 2008; Marazita et al., 2016).

We found a positive correlation between the duration of constant HS to the accumulation of oxidative damage and the increase in SA-β-gal senescence marker staining. During SIPS, cells are known to obtain a SAPS, which in turn can cause collateral damage to neighboring cells and tissues (Zhang et al., 2019). This might explain some of the negative effects of HS treatments and link cellular stress with the physiological distress animals experience in a warm climate. Moreover, this finding might prove useful in the clinic where we wish to have better control of the proliferative status of the cells used for therapy. Our data suggest that thermal shock may have physiological consequences on tissue homeostasis, which could further lead to organ damage and the development of inflammatory and age-related diseases.

Based on existing knowledge about MSCs and senescence, the rate and manifestation of replicative senescence in human and bovine MSCs are in remarkable agreement (Wagner et al., 2008; Gnani et al., 2019; Wiese et al., 2019). However, the effect of hypoxia (Antebi et al., 2018) and thermal stress (Choudhery et al., 2015; Andreeva et al., 2016; McClain-Caldwell et al., 2018; Wang et al., 2019) were inverted; i.e., hypoxia was destructive in human but not in bovine MSC, while mild HS delayed replicative senescence in human. This implies intriguing differences between organisms or tissues of origin as most human MSC studies are carried out on bone marrow or adipose MSCs. This might be an important issue for further study since, even though MSCs have been widely used for the treatment of companion animals, little is known about their potential in the livestock industry (Hill et al., 2019).

Although not yet fully understood, MSCs are thought to play an instrumental role in the maintenance of the body homeostasis. Senescent cells accumulate with age or exposure to stress, cause chronic inflammation, and increase the risk of many diseases (Kornienko et al., 2019). Senescence-related MSC failures, including immunomodulatory activity, hematopoiesis, and paracrine regulation, were shown in human (Munoz-Espin and Serrano, 2014; Li Y. et al., 2017). Understanding the effect of common environmental stress on the functioning of these cells will enhance our knowledge of the consequences of stress at the animal level. Presently, when air temperature in many parts of the world is increasing during the summer months, the study of short- and long-term effects of heat stress on physiological conditions is becoming increasingly important.

## Data Availability Statement

All datasets presented in this study are included in the article/Supplementary Material.

## Author Contributions

ChS conceived and designed the study, designed and performed the experiments, analyzed and interpreted the data, and wrote the manuscript. MG established the experimental system. IS, IR-C, DN, and CaS collected the experimental data and prepared the manuscript. SS conceived and designed the study, assembled and analyzed the data, and wrote the manuscript. All authors contributed to the article and approved the submitted version.

## Conflict of Interest

The authors declare that the research was conducted in the absence of any commercial or financial relationships that could be construed as a potential conflict of interest.

## Supplementary Material

Supplementary Table 1 List of all mouse primers used in this study for gene expression analysis by RT-qPCR.

Supplementary Table 2 List of all bovine primers used in this study for gene expression analysis by RT-qPCR.

Supplementary Table 3 List of first and secondary antibodies used in this study.
