# Supplementary material for: Heat Shock Alters Mesenchymal Stem Cell Identity and Induces Premature Senescence
Source: Front Cell Dev Biol. 2020 Sep 22;8:565970. doi: 10.3389/fcell.2020.565970 (PMC7537765; doi:10.3389/fcell.2020.565970)
Supplement: Supplementary file 1 [file Data_Sheet_1.pdf]

Supplementary Materials and Methods

Umbilical cord (UC) tissue was obtained from two sources, at birth from dairy cows at the research dairy farm in the Israeli agricultural research organization – Volcani center, and from fetuses obtained from abattoirs located in the north of Israel. Ethics approval has been granted. MSC characterization and immunomodulatory potential were assessed using bUC-MSC from different sources, while following HS experiments were carried out on one source only.

Tissue processing and cell culture

The UC tissue was processed following Toupadakis et al., (37). In short, the UC was first soaked in 70% ethanol for 30 seconds and then soaked in Dulbecco's phosphate-buffered saline (DPBS) containing 3% Penicillin-Streptomycin), 1% Gentamycin and 1% Amphotericin b to prevent contamination further down the line. Blood vessels were removed from the tissue, the tissue was minced into small pieces and incubated in a 37 °C water bath for 1 hour in a digestion cocktail containing 0.4% (w/v) Collagenase Type I, 0.4% (w/v) Collagenase Type II, 0.008% (w/v) Elastase Type IV, and 0.2% (w/v) Hyaluronidase. Following digestion, cells were separated from the remaining tissue by filtration through a 70μm nylon filter and washed several times by centrifugation in DPBS. Cells were plated in a low glucose Dulbecco's Modified Eagle's Medium (Gibco) containing 10% fetal bovine serum (FBS) and a penicillin-streptomycin mixture (5%), expanded and cryopreserved at different passages. The media was changed every 3-4 days and all cells were cultured in a humidified incubator with a controlled environment of 5% CO2 and a temperature of 37 °C, unless mentioned otherwise.

Cell characterization

bUC-MSCs were phenotypically characterized, looking for a fibroblast-like morphology of elongated spindle shaped cells. The number of passages suitable for each cell type was determined

by changes in morphology and specific MSC marker characterization by RT-qPCR. MSC markers were also evaluated using flow cytometry and a fluorescent microscope.

Population doubling (PD) time assessment

Following pulse and constant heat shock treatments, bUC-MSCs were trypsinized, counted and 100K cells from each treatment were plated in 10-cm plates. This process was repeated every 4-6 days, for 15 passages (over 100 days). PD and PD time were calculated using the formulas $N=N0\times2d$ (where N, N0 and d are the final cell number, the initial cell number and the number of cell divisions, respectively) and $N=N0\times2t/\tau$ (where N, N0 and τ are the final cell number, the initial cell number and population doubling time, respectively).

Colony-Forming-Unit Fibroblast assay (CFU-F)

Following pulse and constant heat shock treatments, one hundred bUC-MSCs from each treatment were plated on 10-cm dishes in growth medium and cultured for 7 days with medium changes every 3 days. At the end of day 7, colonies were rinsed with phosphate-buffered saline (PBS), fixed with 0.5% crystal violet dye in methanol for 30 minutes at RT, and again rinsed several times with PBS to remove residual dye. After plates were completely dry, colonies were counted and analyzed. 3 biological repeats were used for each treatment.

RNA extraction ,reverse transcription, and Real-time PCR

RNA extraction from cells was carried out using PureLink RNA Midikit (Invitrogen). RNA was then reverse-transcribed into cDNA using High-Capacity cDNA Reverse transcription kit (Applied Biosystems, Waltham, MA, USA). Real-Time PCR (RT-qPCR) reactions were performed using Fast SYBR Green Master Mix (Applied Biosystems) in an ABI Step-One Plus Real-Time PCR system. To ensure validity, each sample tested in triplicates (technical replicates). All primers used were tested and found agreeable with standard curve evaluation and are shown in the primer table (Supplementary Table S1 and S2). The relative mRNA fold change was calculated with the ΔΔct

method using 1-2 control genes as reference or is given as a fold change from control, without normalization.

Flow cytometry

bUC-MSCs were detached from the plate using trypsin solution then filtered through a 35μm nylon filter (Thermo Fisher, Waltham, MA, USA), into Falcon FACS test tube (Thermo Fisher). The fluorescence of the cells was determined using the Flow Cytometer Cytoflex from Beckman Coulter (Brea, CA, USA), equipped with 488nm laser. FL1 (FITC-A) and FL2 (PE-A) channels were used to determine green or red fluorescence, respectively. A minimum of 10,000 cells was examined for each measurement at a flow rate of 12-35μL/second. Broken cells were excluded from the analysis utilizing gating based on cell complexity and cell size (SSC-A and FSC-A respectively) parameters. Fluorescent positive cells were gated using a negative (undyed) cell sample used to determine basal/autofluorescence of the cells. Flow cytometry results were analyzed using FlowJo V.10 software and data is shown as geometric mean intensity or as % of gated cells.

Expression of MSC surface markers

The expression of MSC surface protein CD44 and CD73 was determined using flow cytometry. Cells were collected using a cell scraper, resuspended and counted. Prior to the incubation with the antibodies, for FC blocking, the cells were incubated in PBS containing 2% bovine serum albumin for 1 hour followed by a 1-hour incubation with the appropriate primary antibodies, with approximately $1 \cdot 10^6$ cells analyzed for each antibody, in PBS containing 2% FBS. The cells were then incubated with a suitable secondary antibody as is shown in Supplementary Table S3 and analyzed by flow cytometry. Cells were washed twice following each incubation and all steps were performed on ice, using cold PBS. Adapted from (38).

Cell counting

For heat shock treatments cell count, 40K bUC-MSCs were plated on 6-well plates. On the next day both constant and pulse HS treatments began and on day 3 cells were trypsinized and counted. Counting was performed by mixing 6µL of cells suspended with 6µL Trypan-blue and subsequently loading into an appropriate cell-counting slide. Cells were counted using TC20™ automated cell counter (Bio-Rad Laboratories Hercules, CA, USA). 3 biological triplicates were used for each experiment.

Evaluating immunomodulation function

MSCs anti-inflammatory properties were examined through their ability to suppress the immune response, testing their effect on T cell proliferation using a mixed lymphocyte reaction (MLR), adapted from Arzi et al. (39) and examining phenotypic shifts in macrophages towards their anti-inflammatory state (M2), adapted from (23-25).

T cell proliferation

Blood samples were taken from the jugular vein from dairy cows at the research dairy farm in the Israeli agricultural research organization – Volcani center. The PBMCs were isolated from whole blood using Histopaque 1077 and Histopaque1119 (Sigma). PBMCs were then either cryopreserved or plated for a T cell proliferation assay. The assay was composed of four treatments, activated and non-activated PBMCs and activated and non-activated PBMCs co-cultured with bUC-MSCs at a ratio of 5:1, respectively. Concanavalin A (Sigma-Aldrich) was added for T cell activation on day one of the assays at a concentration of 5µg/mL. On day four, to test cell proliferation, Methylthiazolyldiphenyl-tetrazolium bromide (MTT) was added at 5µg/mL for the final 6 hours of incubation. The amount of MTT formazan produced during the incubation was measured using SpectraMax i3 plate reader (Molecular Devices, San Jose, CA, United States) at a wavelength of

550nm. It should be noted that no purification of T cells was done, however, bovine PBMC are reach with T cells, and Con A is a well-known T cells activator.

Co-culture of bUC-MSCs and RAW macrophages

bUC-MSCs were co-cultured with RAW macrophages to test their anti-inflammatory properties. The experiment consisted of four different treatments, activated and non-activated RAW macrophage and activated and non-activated RAW macrophage co-cultures with bUC--MSCs. Regarding heat shock experiments, on day 3 following pulse and constant HS treatments, co-culture with RAW was initiated. For the co-culture, RAW macrophages were added to the bUC-MSCs at a ratio of 5:1, respectively. 21 hours afterward, activation of RAW macrophages towards M1 was performed using a 1μg/mL of lipopolysaccharides (LPS) in RPMI media for 3 hours. Once activation was over, cells were harvested from plates using cell scrapers and were taken for several analyses to evaluate the macrophages' phenotypic shift, including M1 and M2 markers expression using flow cytometry and gene expression analysis using RT-qPCR.

Macrophages phenotypic shift analysis using M1/M2 markers

Following RAW macrophages co-culture with bUC-MSCs and activation, cells were harvested and were incubated with antibodies specific for CD86, which was used as an M1 marker, and CD163, which was used as an M2 marker. The cells were incubated in PBS containing 2% bovine serum albumin for 1 hour followed by a 1-hour incubation with primary antibodies in PBS containing 2% FBS. The cells were washed and incubated with a suitable secondary antibody (supplementary Table S3), following by analysis by flow cytometry. Alternatively, mRNA was extracted for RT-qPCR with mouse specific primers for M1 and M2 cytokine expression pattern.

Differentiation potential assessment

The multipotency of MSC was examined by testing their multi-lineage differentiation potential into osteoblasts, adipocytes, and chondrocytes. Regarding heat shock experiments, on day 3 following

pulse and constant HS treatments, differentiation protocol was initiated. To induce osteogenic differentiation, cells were plated in 24-well plates and cultured with differentiation media containing LG-DMEM, 10% FCS, 10^{-7}M dexamethasone, 100μM ascorbic acid-2-phosphate and 10mM β –glycerophosphate, following Gomez-Leduc et al. (40). Media was changed every 3 days and following 10 days osteogenic differentiation was assessed using alizarin red S staining. For adipogenic differentiation, the cells were plated in 24-well plates and cultured in LG-DMEM, 10% FBS, 10^{-6}M dexamethasone, 0.5mM 3-isobutyl-1-methyl-xanthine, 0.2mM indomethacin, and 10μg/mL recombinant human (rh) insulin (40). Adipogenic differentiation was assessed by staining with 0.3% oil red O in isopropanol or with 4,4-difluoro-1,3,5,7,8-pentamethyl-4-bora-3a,4a-diaza-s-indacene (Bodipy 493/503, Invitrogen), added with 5μg/mL DAPI (Sigma). To promote chondrogenic differentiation, cells were plated in 24-well plates and cultured for 3 weeks in Dulbecco's Modified Eagle Medium:Nutrient Mixture F-12 (DMEM/F-12) media supplemented with 5% FBS, 100 IU/mL penicillin, 0.1mg/mL streptomycin, 2mM L-glutamine (Biological Industries), 1% Insulin-Transferrin-Selenium (ITS-G, Gibco) and 10ng/mL Transforming Growth Factor-beta 1 (TGFβ1, PeproTech). differentiation was assessed by staining with 0.6% Alcian Blue (Sigma-Aldrich). Following staining, plates were taken to microscopic evaluation, using EVOS FL Auto imaging system (ThermoFisher Scientific). Negative control was carried out using stained cells grown without differentiation media. Adapted from (41, 42).

Immunofluorescence

For MSC's markers assay, cells were grown on microscope coverslips inside a 24-well tissue culture plate for 1-2 days before IF protocol took place. For phosphorylated histone H2AX (Ɣ-H2AX) detection following HS experiments, 2500 bUC-MSCs were plated on microscopic coverslips inside a 12-well plate, on the next day both pulse and constant heat shock treatment began, and on day 3 IF protocol was carried out. All cells were washed once with PBS and fixated with 300μL 4%

paraformaldehyde for 10 minutes at RT. After fixation, cells were washed 3 times with PBS and permeabilized with 0.2% Triton for 5 minutes, then washed with PBS. Next, cells were incubated with PBS+1% BSA+0.1% Saponin with first antibody at 4 °C overnight. On the following day, cells were washed 3 times with PBS and incubated with PBS+1% BSA+0.1% Saponin containing 5μg/mL DAPI (Sigma) and second antibody (Supplementary Table S3) for 1 hour at RT in the dark. Negative control was carried out using only secondary antibody in order to minimize background fluorescence. Next, cells were washed 3 times with PBS and then mounted on microscope slides using aqueous fluorescent mounting medium (GBI labs).

Oxidative stress levels detection

To measure oxidative stress in live cells, CellROX Green Reagent (Invitrogen) was used. After cells were treated according to the relevant HS treatment, on day 3 bUC-MSCs reached a confluence of 80-90%, were washed once with PBS and were then incubated with PBS containing 2% FBS, 0.5μM of CellROX reagent with or without 500μM H2O2 (Sigma) for 30 minutes at 37 °C. Cells were then washed twice with PBS, trypsinized and filtered to flow cytometry tubes, followed by green fluorescence measurement using flow cytometry. 3 biological repeats were used for each treatment.

Mitochondrial membrane potential measurement

In order to measure mitochondrial membrane potential ($\Delta\Psi m$), following pulse or constant heat shock treatments, bUC-MSCs reached 80% confluency and were trypsinized and suspended in 1mL warm media containing 5μg/mL of 5,5′,6,6′-tetra-chloro-1,1′,3,3′-tetraethylbenzimidazolyl carbocyanine iodide fluorescent probe (JC-1; ENZO life sciences international) for 25 minutes at 37 °C. Next, cells were centrifuged and washed twice with PBS and re-suspended in PBS. Cells were then taken to flow cytometry, measuring both green (FL1/FITC-A) and red (FL2/PE-A) fluorescence. Mitochondrial membrane potential was qualified based on the fluorescence emitted and classified into two main colors: red – high potential and green – low potential. 3 biological repeats were made

for each treatment and a one-hour pre-staining incubation with 500µM H_2O_2 was made for positive control.

Cell death quantification

For quantification of cell death in culture, cells were trypsinized, washed twice with PBS and re-suspended with 10µg/mL of propidium iodide for 5 minutes on ice. Next, cells were taken to flow cytometry for red fluorescence detection. A positive control was taken following a 4-hour incubation with 1µg/mL of Staurosporine pre-staining.

Analysis of apoptosis levels

For measurement of apoptosis levels in culture, cells were trypsinized, washed with PBS and then re-suspended with annexin-binding buffer (containing 10 mM HEPES, 140 mM NaCl, and 2.5 mM $CaCl_2$). 1:20 Annexin V (Invitrogen) was added and incubated for 15 minutes at RT. Next, samples were added with annexin-binding-buffer and taken to flow cytometry for green fluorescence detection. Positive control was taken following a 4-hour incubation with 1µg/mL of Staurosporine pre-staining.

Cell proliferation assay

BUC-MSCs were plated for different treatments. On the next day, cells were stained using PBS containing 5µM of reagent from CellTrace CFSE Cell Proliferation Kit (Invitrogen) for 20 minutes at 37 °C. Cells were then washed twice with PBS containing 2% FBS and culture media was added. 3 days afterward cells reached confluence of 80-90%, positive control was made using the same protocol, and a negative (no dye) control was also taken. All cells were trypsinized, filtered to flow cytometry tubes and were taken to flow cytometry, measuring their green fluorescence. 2-3 biological repeats were used for each treatment.

Cell cycle analysis

Following pulse and constant heat shock treatments, 1 million bUC-MSCs were incubated with 30μM Bromodeoxyuridine (BrdU) for 1 hour at 37 °C. Next, cells were washed once with PBS, trypsinized and centrifuged for 2 minutes at 3000rpm. Cells were then washed with PBS and fixated using 70% ice-cold EtOH and stored at 4°C for at least 30 minutes. Following fixation, cells were centrifuged and re-suspended in denaturing solution containing 2M HCL/0.5% Triton x-100 for 30 minutes at RT. Next, cells were centrifuged and washed with wash buffer (0.5% BSA in PBS). Cells were then re-incubated in 0.1M Sodium Tetraborate for 2 minutes at RT. Next, cells were washed with wash buffer and centrifuged, following by first antibody incubation for 1 hour at RT containing 0.5% Triton x-100/0.5% BSA in PBS, and anti-BrdU antibody (Supplementary Table S3). Following first antibody incubation, cells were washed with wash buffer, and re-suspended with 0.5% Triton x-100/0.5% BSA in PBS added with appropriate secondary antibody for 1 hour at RT in the dark. Following second antibody incubation, cells were washed with wash buffer and incubated with PBS containing 10μg/mL RNAse A (Pure Link) and 50μg/mL Propidium Iodide (PI, Sigma) for 1 hour at RT in the dark. 2 negative control samples were taken using only PI or anti-BrdU with secondary antibody in order to minimize background fluorescence. Following incubation, cells were taken to flow cytometry, measuring both red and green fluorescence for full cell cycle analysis and S phase quantification, respectively. 3 biological repeats were used for each treatment.

For detection of S positive cells during the 43 hours prior to the end of HS treatments, cells from 42 ^{o}C pulse HS and 72h constant HS were incubated with BrdU for a range of time periods (1-43 hours) and taken for BrdU detection protocol on day 3. The protocol was carried out as described and following first and secondary antibodies cells were taken to flow cytometry for S phase quantification. 3 biological repeats were taken for each treatment and negative control was taken without BrdU but with first and secondary antibodies.

BrdU assay for cell-cycle S phase quantification

BrdU immunohistochemistry was performed as described previously (Lichter et al. 1990; Selig et al. 1992). Following pulse and constant heat shock treatments, 1 million bUC-MSCs were incubated with 30μM Bromodeoxyuridine (BrdU) for 1 hour at 37°C. Next, cells were washed with PBS, trypsinized and centrifuged. Briefly, cells were treated for isolating nuclei using hypotonic KCl (0.5%) treatment and were fixed with Methanol: Acetic acid (3:1). Following fixation, denaturation was carried out by incubation in 70% Formamide and 2× SSC for 2 min at 72 °C, then slides were dehydrated by a series of ice-cold Ethanol washes (70%, 90%, and 100% for 5 min each). BrdU was detected by an anti-BrdU antibody followed by FITC-conjugated anti-mouse antibody (supplementary Table S3). Following the first and second antibody, cells were mounted on slides and BrdU positive cells were counted using fluorescent microscopy.

Senescence-associated β-galactosidase marker assay

Following pulse and constant heat shock treatments, bUC-MSCs reached 80%-90% confluency and were incubated with 0.15μL of dye per 500μL media for 1 hour at 37 °C, using Senescence Assay Kit (Abcam). Cells were then washed twice with wash buffer, trypsinized, suspended in wash buffer and taken to flow cytometry, measuring green fluorescence. 3 biological repeats were used for each treatment.

Supplementary Figures

Supplementary figure S1

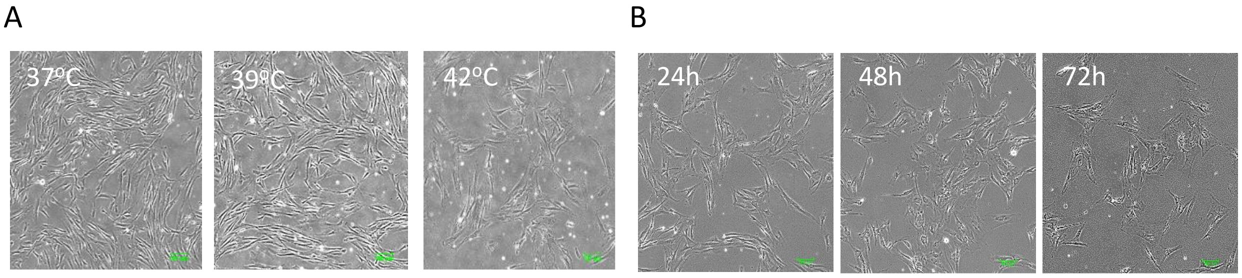

Figure S1: morphology of bUC-MSC changes following HS treatments.

Microscopic evaluation of bUC-MSC morphology. Larger, flattened cells were evident following 42 °C pulse (A) and 48h, 72h constant (B) HS treatments. Scale bar= 100μm.

Supplementary figure S2

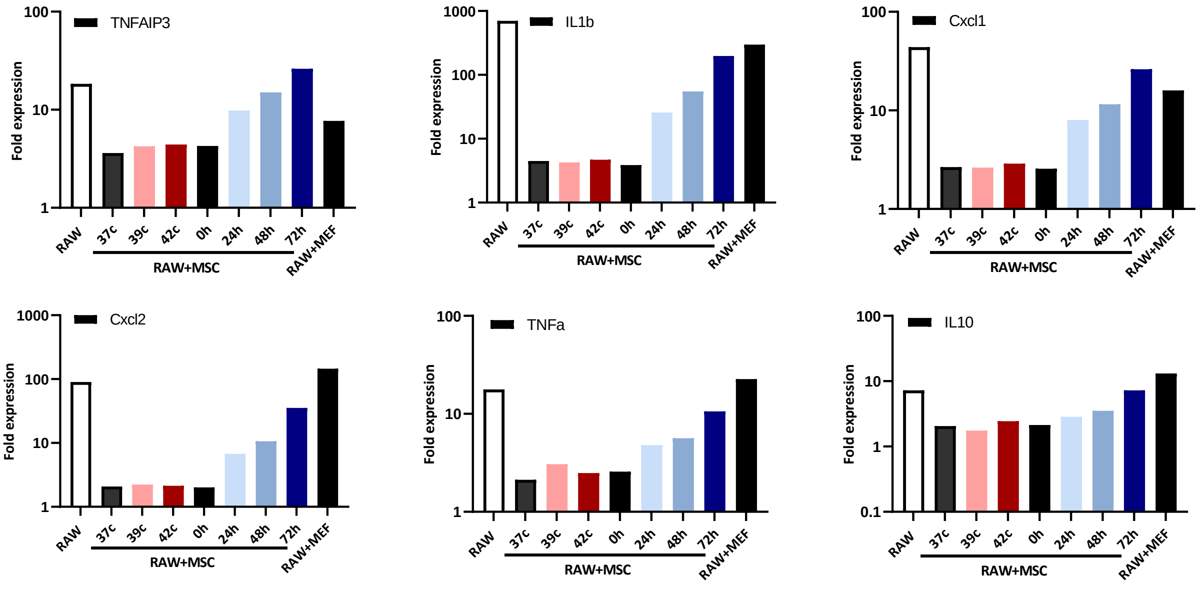

Figure S2: loss of immunomodulatory function following constant heat shock.

The transcriptional profile of neutrophil recruitment (using Ly6G) and inflammatory mediators (CXCL2, CXCL1, IL6, IL1β, TNFα, iNOS) genes were quantified using RT-qPCR. The expression levels of those typical pro-inflammatory genes were high in RAW cells alone (white) and reduced in RAW cells co-cultured with bUC-MSC but not with MEFs. Long HS treatment at 40.5 °C eliminates the reduction, indicating those cells are malfunctioning. Three housekeeping genes were used as reference genes – UBC, EIF5a and EEF1A1. Data are mean ± SEM for n=3.

Supplementary figure S3

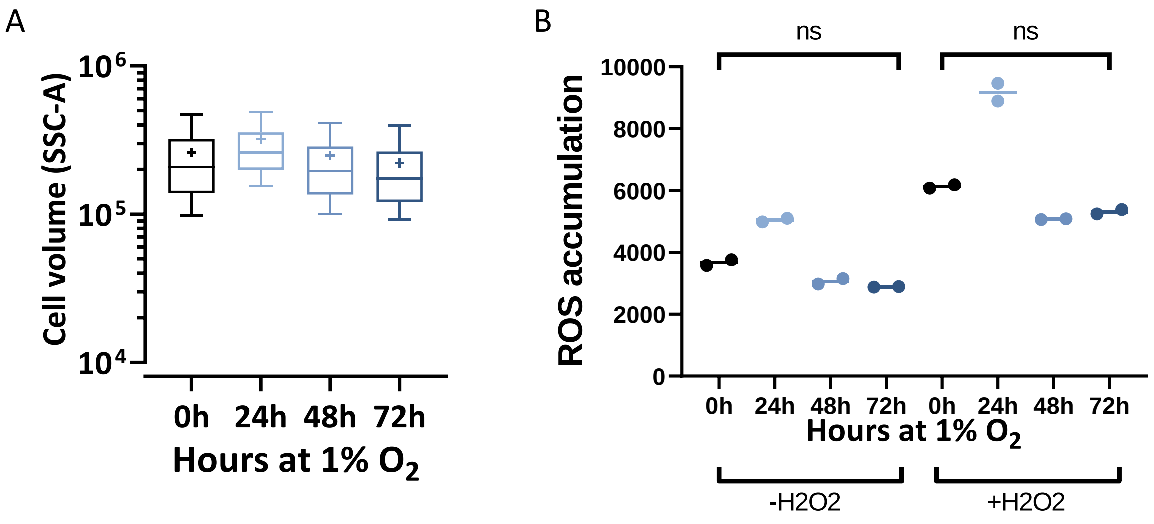

Figure S3: effect of hypoxia on bUC-MSCs' cell size and ROS accumulation.

(A) Side scatter geo-mean data of cells following different incubation hours at 1% O_2 are shown. One flow-analysis of 10,000 cells is shown (representative result, n=3). (B) Flow cytometry results of CellROX staining for cellular ROS levels without and with 30 min. 500µM H_2O_2, for cells incubated at 1% O_2 for different hours. Data presented is a mean of green fluorescence, ± SD, n=2.

Supplementary figure S4

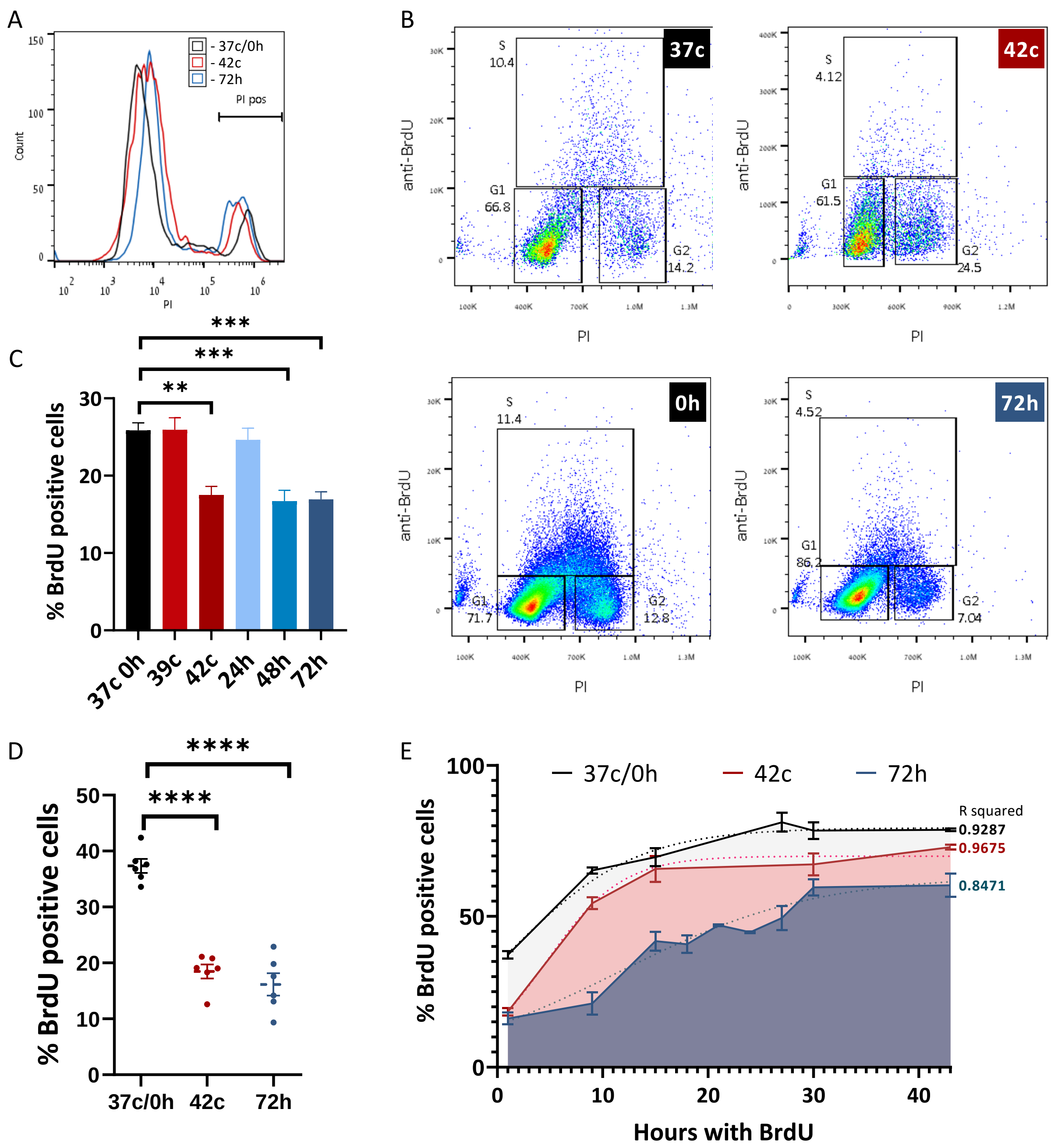

Figure S4: HS treatments do not result in major apoptosis rate but rather in cell cycle arrest

(A) Representative flow cytometry analysis of PI positive staining for control (black), pulse (red) and constant (blue) apoptosis rates. (B) PI-BrdU flow cytometry representative results of cell cycle analysis for control (0h, 37c), constant (72h) and pulse (42c) HS treatments. (C) Quantification of S-phase cells was carried out using BrbU antibody and positive cells were counted using fluorescent

microscopy. Percentage of BrdU positive cells from total cells is presented. Statistical analysis was performed using chi-squared-test. More than 400 cells were counted. (D) Quantification of S-phase cells was carried out using BrbU antibody and positive cells were counted using Flow cytometry. Statistical significance determined using the Holm-Sidak method, with alpha = 0.05. (E) Detection of BrdU incorporated for 1-43 hours into the DNA of pulse HS at 42 °C and constant HS for 72 hours treated cells by flow cytometry. Data are mean ± SEM for n=3-6. Logistic growth R squared fit is shown.

Supplementary figure S5

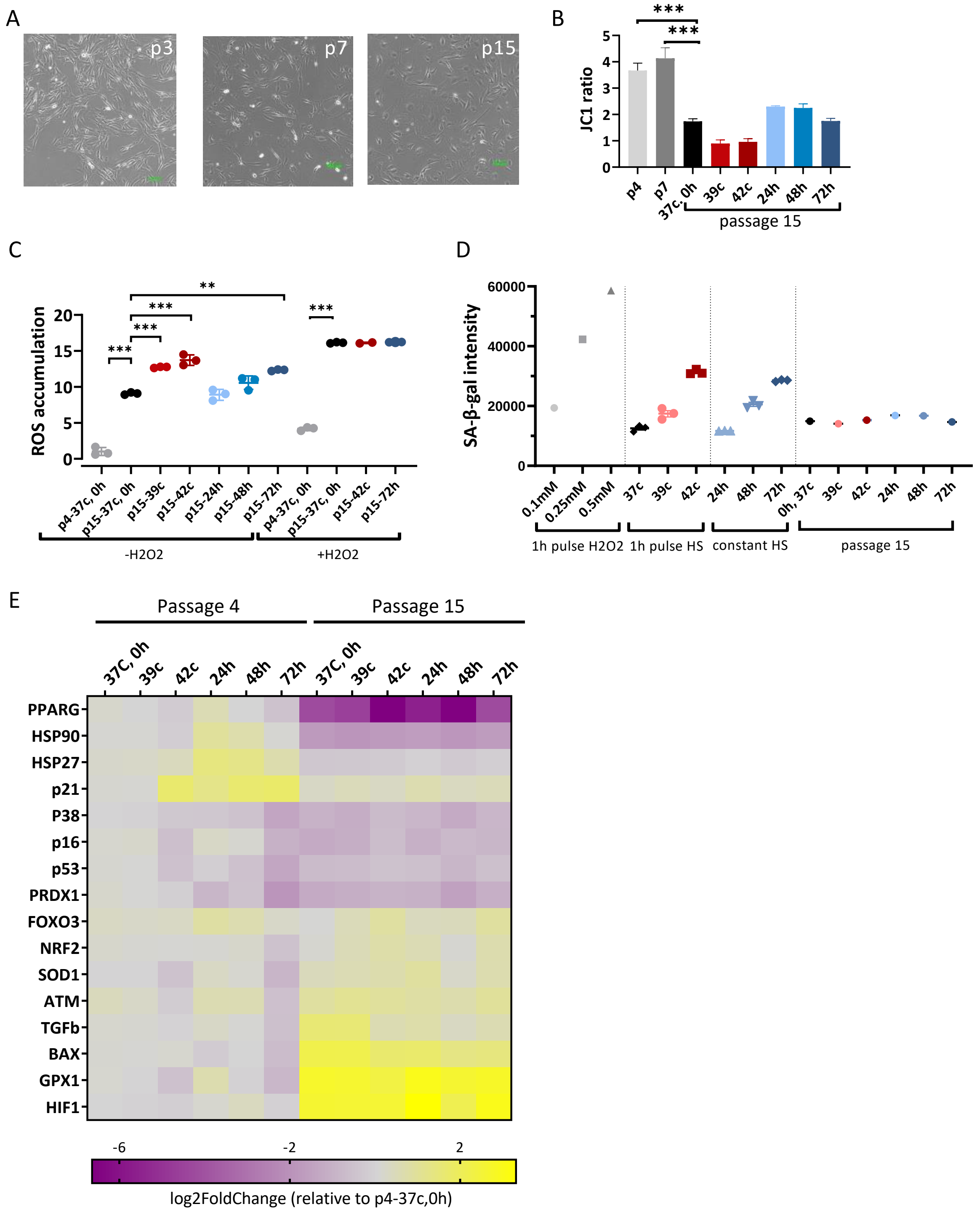

Figure S5: cells from late passage show different cellular damage signals than cells after HS.

(A) MSC display changes in cell morphology following multiple passages (P3, P7 and P15). Cells display a less-spindle shaped morphology and fewer sharp edges, forming abnormal formations.

Scale bar = 100µm. (B) Mitochondrial membrane potential for multiple passages was measured using JC1 assay and flow analysis. High JC1 ratio of aggregate (red)/monomer (green) indicates high mitochondrial function, while low ratio is a sign of mitochondrial damage. n=3. Statistics: Tukey multiple comparison test, ***$p<0.0001$. (C) Flow cytometry results of CellROX staining of P4 cells and P15 cells after HS treatments. P4 untreated (gray) cells show the lowest amount of ROS without and with H_2O_2 while HS treated and untreated P15 cells show higher ROS levels. Data presented is a geometric mean ±SD of green fluorescence, n=3. Tukey multiple comparison test was performed, **$p<0.001$, ***$p<0.0001$. (D) SA-β-gal intensity is presented for P4 and P15 untreated cells (black), pulse HS cells (red) and constant HS cells (blue), compared to SA-β-gal intensity of cells treated for 1 hour with different concentrations of H_2O_2, 48 hours pre-staining. P15 cells showed no differences between HS treatments, but higher SA-β-gal intensity than P4 untreated, 37 °C and 24H ones (n=3 for P4 cells, n=1 for P15 cells). (E) Heat map showing changes in expression levels for the indicated genes in untreated (37c, 0h), pulse HS (39c, 42c) or constant HS (24h, 48h, 72h) treatments, passage 4 and 15 MSC. Fold changes were calculated and color-coded relative to the values in the P4 control (0h, 37 °C for 3 days) cells.
